# Impact of telephone nursing education program for equity in healthcare

**DOI:** 10.1186/s12939-016-0447-0

**Published:** 2016-09-21

**Authors:** Anna T. Höglund, Marianne Carlsson, Inger K. Holmström, Elenor Kaminsky

**Affiliations:** 1 Department of Public Health and Caring Sciences, Box 564, SE-751 22 Uppsala, Sweden; 2 University of Gävle, 801 76 Gävle, Sweden; 3 School of health, care and social welfare, Mälardalen University, Box 883, 721 23 Västerås, Sweden

**Keywords:** Equity in health, Intersectional perspective, Intervention study, Telephone nursing, Sweden

## Abstract

**Background:**

The Swedish Healthcare Act prescribes that healthcare should be provided according to needs and with respect for each person’s human dignity. The goal is equity in health for the whole population. In spite of this, studies have revealed that Swedish healthcare is not always provided equally. This has also been observed in telephone nursing.

Therefore, the aim of the present study was to investigate if and how an educational intervention can improve awareness of equity in healthcare among telephone nurses.

**Methods:**

The study had a quasi-experimental design, with one intervention group and one control group. A base-line measurement was performed before an educational intervention and a follow-up measurement was made afterwards in both groups, using a study specific questionnaire in which fictive persons of different age, gender and ethnicity were assessed concerning, e.g., power over one’s own life, quality of life and experience of discrimination. The educational intervention consisted of a web-based lecture, literature and a seminar, covering aspects of inequality in healthcare related to gender, age and ethnicity, and gender and intersectionality theories as explaining models for these conditions.

**Results:**

The results showed few significant differences before and after the intervention in the intervention group. Also in the control group few significant differences were found in the second measurement, although no intervention was performed in that group. The reason might be that the instrument used was not sensitive enough to pick up an expected raised awareness of equity in healthcare, or that solely the act of filling out the questionnaire can create a sort of intervention effect. Fictive persons born in Sweden and of young age were assessed to have a higher Good life-index than the fictive persons born outside Europe and of higher age in all assessments.

**Conclusion:**

The results are an imperative that equity in healthcare still needs to be educated and discussed in different healthcare settings. The intervention and questionnaire were designed to fit telephone nurses, but could easily be adjusted to suit other professional groups, who need to increase their awareness of equity in healthcare.

## Background

The Swedish Healthcare Act (SFS 1982:763) prescribes that healthcare should be provided according to needs and with respect for each person’s human dignity. The goal is equity in health for the whole population. In spite of this intention, studies have revealed that healthcare in Sweden is not always provided equally. Primarily, gender inequalities have been reported [1, 2]. It has also been reported that there are gender related patterns in people’s healthcare seeking behaviour. For example, women tend to seek care more actively than men, and they are also more eager to contact healthcare services on behalf of others, not least their children and partners [3–5].

Such patterns have also been observed in telephone nursing. This is a service which has developed rapidly in many Western countries during the last decades. In Sweden, the service Swedish Healthcare Direct (SHD) 1177 was launched in 2013, and all regions and county councils in the country are currently connected to the service, through the telephone number 1177 on a 24/7/52 service basis. It is meant to be citizens’ first healthcare contact and is staffed by 1500 registered nurses (RNs) located at 33 sites. These nurses answer about 5.5 million calls yearly (http://www.1177.se/), making SHD one of Sweden’s largest healthcare providers. Similar services can be found in, e.g., the UK, Australia and the US [6].

The intention behind SHD is to make healthcare more efficient, accessible and safe for patients (http://www.1177.se/). Telephone nurses in Sweden are encouraged to handle six to eight calls per hour [7]. An important task for telephone nurses is to steer patients to an appropriate care level. Further, their tasks are to assess symptoms, recommend a doctor’s appointment or give self-care advice [8].

By tradition, Swedish healthcare is tax financed. For the past 20 years, however, the system has undergone key changes within the neoliberal realm, with New Public Management (NPM) reforms and privatization at its centre [9, 10]. NPM is a management ideology used in the public sector, with origin in the private sector. It is characterised by demands of efficiency, cost control and performance evaluation [11], and consequently often used as a control system. The goal is to become more market oriented, by holding public institutions accountable for their work performance and base resource allocation on performance. Thus, one of the core issues is that of performance measurements. Studies have reported the influence of NPM also in telephone nursing services [12], which is considered to be a cost-effective way to provide healthcare [13]. Some of the SHD call centres are privately run. Hence, telephone nurses work in a call centre-like milieu, and efficiency and profit are guiding concepts [14].

Telephone nursing has been argued to have the potential to increase equity in health, as it is easy to reach and open to everyone [7, 15, 16]. However, studies have reported that gender can influence the use of the service. Not least, telephone nursing has been described as a female service, in that it is both handled and used mainly by women [4]. About 90 % of the Swedish work force of telephone nurses consists of women [17] and a majority of the callers are women [16]. Likewise, a Swedish study found that in paediatric health calls a majority of the callers were mothers [5]. In order to understand these patterns more in depth, a Swedish study explored the communication between telephone nurses and callers, using Critical Discourse Analysis (CDA). The discourse of telephone nursing was found to be dialectically related to neoliberal ideology, such as NPM, and the ideology of medicine, as well as situated in a gendered context of ideal femininity and masculinity [15].

Concerning male callers, telephone nurses have described them as either assertive or reluctant, while female callers were perceived as easier to persuade to “wait and see” [4]. A British study identified three different types of male callers, namely the assertive carer, the reluctant patient and the new dad [18]. Differences have also been reported concerning the advice given by telephone nurses. A Swedish study showed that the likelihood for a father to receive referral to health services from a telephone nurse were twice as great as for mothers [5].

Also ethnicity and age have been pointed out as constituting factors for use of telephone nursing services. Previous studies indicate that ethnical minorities, deprived groups and the elderly tend to underutilize the service [16, 19–23]. Hence, a high awareness of equity in health and how factors such as gender, ethnicity and age can intersect seems to be of outmost importance for nurses working with the service.

Against this background, the aim of the present study was to investigate if and how an educational intervention can improve awareness of equity in healthcare among telephone nurses. Two central questions were investigated:

1. How aware are telephone nurses of equity in healthcare?
2. How can this awareness be impacted by an educational intervention?

### Theoretical framework

The concept of ‘gender’ came into practise in the 1970s, in order to being able to analyse differences between men and women that were not related to the biological sex [24, 25]. In the 1980s, West and Zimmerman [26] introduced the concept “doing gender” and since then researchers within this field mostly refer to gender as something that is constantly constructed in interaction between human beings. Early gender research focused on the construction of femininity, with the aim of understanding and changing women’s subordination in patriarchal societies. Since the 1990s, theories of masculinity have also been developed, for example by Connell [27, 28], who argued that masculinity and femininity are contextually and relationally constructed, so that in the same context, several forms of masculinity (and femininity) can be found, hierarchically ordered in relation to each other. The most valued form of masculinity in a Western context was defined by Connell as ‘hegemonic masculinity’ [27, 29], characterised by being constructed as superior to femininity. Hegemonic masculinity is normative rather than normal; even though few people might live up to these idealized images, men are required to position themselves in relation to them. In analogy with Connell, Schippers [30] identified a “hegemonic femininity”, i.e., the most valued form of femininity in a Western context, which guarantees the dominant position of men and the subordination of women. Further, Lyons [31] has argued that hegemonic femininity is marked by being caring and acting responsibly towards other people.

“Doing health” has shown to be an important aspect of “doing gender” [32]; for example, hegemonic masculinity can be reinforced by men being reluctant to seek healthcare [33–36]. In Western countries, men are likely to die at younger age and suffer more frequently from heart attacks and mortality from cancer than women [37]. Furthermore, men have unhealthier behaviours than women, for instance, through a higher extent of excessive drinking, unsafe sex and taking less health preventive actions [34]. This is also related to a failure or delay in men’s care- seeking when ill. Ideal femininity is upheld by the opposite health behaviour; that is, taking care of one’s own and other family members’ health and seeking healthcare in time [31].

Macintyre and Hunt [38] have pointed out that gender, along with socio-economic factors, as well as ethnicity, age and sexuality, are important markers of how groups and individuals are positioned in a society. This implies that gender is a dimension of social life that interacts – or intersects – with other factors, such as ethnicity, race, age, sexual orientation or identity and social class. This has also been pointed out by Connell [27]. Also within healthcare research, the concept of ‘intersectionality’ has been frequently used and acknowledged [39–42]. Wamala et al. [43] found that perceived discrimination due to more than one social category increased the risk of refraining from seeking healthcare, despite an experienced need.

## Methods

The study had a quasi-experimental design, with one intervention group and one control group. A base-line measurement was performed before the educational intervention and a follow-up measurement was made afterwards in both groups.

### Sample and setting

Telephone nurses from two different sites in Sweden were asked to participate in the study. The sites were strategically chosen, since they were similar in size and in number of employed (30–40) nurses. One site functioned as intervention group and the other as control group, which implies that samples in the same group were from the same working place and that the participants were not blinded to the intervention.

Permission to perform the study was given by the respective head of the sites. All telephone nurses working on the sites were asked to participate and they were included after individual informed consent. All in all, 72 nurses participated in the baseline measurement (32 in the intervention group and 40 in the control group). In the intervention seminar 19 nurses participated. In the follow-up evaluation 60 nurses participated (25 in the intervention group and 35 in the control group). All 25 nurses in the intervention group who answered the follow-up questionnaire were analyzed together, although only 19 of them participated in the seminar that was part of the intervention. This is due to the fact that they had all received information about the literature and had access to the web-based lecture, also those who did not take part in the seminar because of various reasons (illness, work load etc.).

### Instrument

The questionnaire was developed by the research group and is described elsewhere [44, 45]. It measures three aspects of power orders or intersects, namely, gender, age and ethnicity. It included descriptions of twelve fictive persons, of different age and born in Sweden or outside Europe, and of male or female gender. The following fictive persons were included:

- Isa: 25 years, born in Sweden, female
- Lynn: 25 years, born outside Europe, female
- Alexander: 25 years, born in Sweden, male
- Elliot: 25 years, born outside Europe, male
- Johanna: 45 years, born in Sweden, female
- Manuela: 45 years, born outside Europe, female
- Björn: 45 years, born in Sweden, male
- Urghesa: 45 years, born outside Europe, male
- Karin: 70 years, born in Sweden, female
- Li-Xing: 70 years, born outside Europe, female
- David: 70 years, born in Sweden, male
- Ahmed: 70 years, born outside Europe, male

The items concerned assessment of the likelihood of whether the fictive person had called SHD, whether or not s/he was recommended a doctor’s appointment when calling, whether s/he had a high quality of life, power of own life and had experienced discrimination. Each participant was asked to assess two of the fictive persons, randomly selected. This gave 66 different combinations ((12x11)/2). The participants were also asked to give comments to their assessments, using free text.

The base-line questionnaire was distributed to the intervention group and the control group on workplace meetings in fall 2014. The follow-up questionnaire was distributed to both groups on workplace meetings in May and June 2015, i.e., approximately six months after the intervention.

### Description of the intervention

The intervention used can be described as complex, in that it consisted of several interacting components [46]. Complex interventions are characterized by the difficulty of behavior that is required by those delivering and receiving it, as well as by the number of groups and outcomes and the degree of flexibility the intervention permits. Key questions concern whether the intervention is effective in everyday practice, what the active ingredients are and how they are exerting their effect [46]. The interacting components used in the present study intervention were 1) web-based lecture; 2) literature reading; and 3) seminar. Hence, it consisted of reading, listening and reflecting alone and in group settings.

The web-based lecture covered aspects that have been described under “Theoretical framework” above. Based on statistics of inequality in healthcare, Connell’s theory of hegemonic masculinity [27–29] and the concept of “doing gender” [26] in relation to “doing health” [32] were introduced. The literature consisted of three chapters from a Swedish anthology on “gender vertigo” [47] and critical norm theory, edited by the Swedish Association of Health Professionals [48]. The chapters addressed the function of socially constructed norms in society, e.g., gender norms; theories on constructions of masculinity, and research on racism as a result of discriminatory norms.

Flexibility in how the intervention was performed was permitted and the design and performance were based on theory [46]; in this case gender and intersectionality theories. Further, the educational intervention was designed according to principles for adult learning [49]. Knowles [50] first identified the characteristics of adult learning, stressing that it should acknowledge the participants’ autonomy, experience and knowledge. The teaching should be goal oriented, relevant, and practical and the participants be treated with respect in a cooperative learning climate. Further, our intervention also acknowledged principles of the adults’ willingness to learn, that they learn by doing and from practical and realistic problems (http://www.literacy.ca/).

Information of the educational intervention was given both orally and in written to the participants in the intervention group on a workplace meeting in fall 2014. It encouraged them to watch the web-based lecture on inequalities in healthcare and read three chapters in the literature on gender and intersectionality in health.

The seminar was led by two of the authors (ATH and EK) and lasted for about three hours. It was performed twice in fall 2014, covering half the intervention group at a time, in order to fit the work schedule for the telephone nursing site. In the seminar, participants were first allowed to reflect on the web-based lecture and the recommended literature, followed by group discussions on these matters. Thereafter, authentic case examples were discussed in smaller groups. At the end of the session, the participants were asked to fill out a short evaluation form of the educational intervention, covering questions on how participants viewed the literature, the lecture and the seminar.

### Data analysis

Data from the questionnaires were analyzed through descriptive and comparative statistics, using SPSS version 22. The mean (M) was calculated for each assessed person for each item. The assessments were ranked from lowest likelihood to highest likelihood; thus 12 ranked positions were possible. A Good life-index was also calculated. This combined the questions: quality of life, power of own life and experience of discrimination. The assessment for the item “Experience of discrimination” was reversed when the index was calculated. The reliability/homogeneity of the Good life-index was calculated with Cronbach’s alpha-coefficient [51, 52].

### Ethical considerations

Approval for the study was sought at the Regional Ethics Review Board (Dnr 2014/130). The Board found, however, that according to Swedish legislation no formal approval of the project was needed. Throughout the project, the ethics of scientific work as outlined in the Declaration of Helsinki [53] were followed. Permission to perform the study was given by the respective head of the sites. The study concerned the encounter between telephone nurse and caller, with focus on power interactions related to factors such as gender, age and ethnicity; hence, the issue could be regarded as sensitive. In order to handle these ethical challenges, much effort was put on information and the consent process. The nurses were informed both orally and in writing about the study and were included after informed consent. Information emphasized that participation was voluntary and possible to withdraw at any time. Likewise, it was stressed that data would be handled confidentially and that no workplace would be identifiable in the reporting of results.

## Results

Characteristics of the participants before and after the intervention are presented in Table 1.

**Table 1:** Characteristics of the participants of the intervention group and the control group before and after the intervention

	Intervention group				Control group			
	Before intervention (*n* = 32)		After intervention (*n* = 25)		Before intervention (*n* = 41)		After intervention (*n* = 35)	
Age	M*	SD**	M	SD	M	SD	M	SD
*r* = 39–72/34–72 (intervention group); *r* = 42–71/28–67 (control group)	57.28	8.4	57.21	8.1	58.39	6.5	56.09	8.8
Fulltime work	14		6		12		10	
Part time work	17		19 (additional workplace 8)		27		23 (additional workplace 5)	
Retired	1		0		2		2	

*Mean

**Standard deviation

A Good life-index, consisting of assessments of the fictive persons’ possible experience of quality of life, power over own life and the reversed assessment of the question measuring experience of discrimination, was calculated before and after the intervention in both groups. The assessments of the Good life-index for the participants in the intervention group are presented in Table 2 and for the control group in Table 3.

**Table 2:** Good life-index consisting of quality of life, power over own life, and the reversed assessment of experience of discrimination (range 3–18). The Means (M) and Standard deviations (SD) according to the intervention group before (*n* = 32) and after (*n* = 25) the intervention

	Before the intervention				After the intervention		
Rank	Intervention group^a^	M	SD	Rank	Intervention group	M	SD
1	*Karin 70*	12.75	1.5	1	*Johanna 45*	13.67	1.5
2	*Alexander 25*	12.00	1.0	2	*Björn 45*	12.60	2.1
3	*Isa 25*	12.00	2.0	3	*Alexander 25*	12.60	2.7
4	*Björn 45*	11.00	0	4	*Karin 70*	12.50	1.9
5	*Johanna 45*	10.50	1.3	5	*Isa 25*	12.25	1.0
6	Li-Xing 70	10.33	2.9	6	*David 70*	12.20	2.0
7	Elliot 25	10.13	1.6	7	Lynn 25	11.00	2.0
8	Ahmed 70	9.00	1.7	8	Elliot 25	10.00	2.8
9	Urghesa 45	8.11	1.5	9	Ahmed 70	9.50	0.7
10	Manuela 45	8.00	1.5	10	Li-Xing 70	8.40	1.9
11	Lynn 25	7.67	1.4	11	Manuela 45	8.00	2.0
12				12	Urghesa 45	7.67	2.9

^a^There were no assessments concerning David 70 in the measurement before the interventions

Swedish names are presented in italics

**Table 3:** Good life-index consisting of quality of life, power over own life, and the reversed assessment of experience of discrimination (range 3–18). The Means (M) and Standard deviations (SD) according to the control group before (*n* = 41) and after (*n* = 35) the intervention

	Before the intervention				After the intervention		
Rank	Control group	M	SD	Rank	Control group	M	SD
1	*Alexander 25*	12.67	0.6	1	*Johanna 45*	12.50	2.0
2	*Karin 70*	12.50	1.5	2	*Isa 25*	12.40	1.3
3	*Björn 45*	12.33	1.5	3	Manuela 45	12.16	1.7
4	*Isa 25*	12.29	2.4	4	*Björn 45*	12.00	0.0
5	*David 70*	11.50	0.7	5	*Alexander 25*	12.00	2.4
6	Li-Xing 70	11.13	2.4	6	*David 70*	11.70	2.5
7	*Johanna 45*	10.33	1.6	7	*Karin 70*	11.50	1.5
8	Lynn 25	10.33	2.1	8	Lynn 25	11.14	1.3
9	Urghesa 45	9.78	2.3	9	Elliot	9.75	0.9
10	Manuela 45	9.60	1.1	10	Li-Xing 70	9.40	1.5
11	Elliot 25	9.12	2.5	11	Urghesa 45	8.25	2.4
12	Ahmed 70	8.56	1.8	12	Ahmed 70	6.50	2.4

The fictive persons born in Sweden were assessed to have a higher Good life-index in all four assessment situations. Gender, ethnicity and age are three obvious characteristics of a person. If we compare the most different positions of the fictive persons in our questionnaire, we have the following options:

- Gender: female – male
- Age: young – middle aged – old
- Ethnicity: born in Sweden - born outside Europe

Based on statistics [54] and societal norms [28, 29], the most favorable position is supposed to be a young man born in Sweden and the least favorable position an old woman born outside Europe; in this study represented by Alexander and Li-Xing. There was no significant difference in the assessments of Alexander and Li-Xing in the intervention group before the intervention. (See Table 2 for Means (M) and Standard deviations (SD)). (t = 1.34, df = 10, *p* > 0.10; non-significant). After the intervention there was a significant difference between the assessments of Alexander and Li-Xing (t = 2.56, df = 8, *p* < 0.05). The pattern was the same also in the control group, (see Table 3 for Means (M) and Standard deviations (SD)), i.e., no difference before the intervention (t = 1.50, df = 9, *p* > 0.10, non-significant), but a significant difference after the intervention (t = 2.22, df = 11, *p* < 0.05).

Concerning the Good life-index, comparisons were performed, where the fictive persons born in Sweden were compared with the persons born outside Europe. Swedish ethnicity was assessed to give a higher Good life-index than a non-Swedish ethnicity. This pattern was found in the intervention group as well as in the control group, both before and after the intervention (Tables 4 and 5).

**Table 4:** Aggregated Good Life-index before and after the intervention

Intervention group									
Before the intervention					After the intervention				
Swedish names		Non Swedish names		p	Swedish names		Non Swedish names		p
M*	SD**	M	SD		M	SD	M	SD	
11.65	0.9	8.87	1.1	0.000	12.72	0.5	9.10	1.3	0.000
Women		Men			Women		Men		
M	SD	M	SD		M	SD	M	SD	
10.21	2.1	10.01	1.5	0.056	10.97	2.2	11.38	1.5	0.553

**Table 5:** Aggregated Good Life-index before and after the intervention

Control group									
Before the intervention					After the intervention				
Swedish names		Non Swedish names		p	Swedish names		Non Swedish names		p
M	SD	M	SD		M	SD	M	SD	
11.93	0.9	9.75	0.9	0.000	12.01	0.4	9.53	2.0	0.014
Women		Men			Women		Men		
M	SD	M	SD		M	SD	M	SD	
11.03	1.2	10.67	1.7	0.230	11.52	1.6	10.03	2.3	0.029

Comparisons concerning Good life-index were also made between the assessments of the fictive persons who were supposed to be women and those who were supposed to be men. No significant differences were assessed between the fictive women and men in the intervention group (Table 4).

Likewise, comparisons between the fictive persons of different ages showed no statistically significant differences (Tables 6 and 7).

**Table 6:** Aggregated Good Life-index before and after the intervention

Intervention group													
Before the intervention							After the intervention						
Young		Middle-aged		Old		p	Young		Midlle-aged		Old		p
M	SD	M	SD	M	SD		M	SD	M	SD	M	SD	
11.38	1.1	9.87	1.5	10.69	1.9	0.450	11.46	1.2	10.49	1.5	10.65	1.0	0.271

**Table 7:** Aggregated Good Life-index before and after the intervention

Control group													
Before the intervention							After the intervention						
Young		Middle-aged		Old		p	Young		Midlle-aged		Old		p
M	SD	M	SD	M	SD		M	SD	M	SD	M	SD	
11.10	0.8	10.51	0.63	9.77	1.2	0.643	11.32	0.6	11.23	1.0	9.78	1.2	0.09

*Mean

**Standard deviation

The participants were also asked how likely they assumed that it was that the fictive persons had called SHD and in Tables 8 and 9 the results are presented.

**Table 8:** Likelihood of having called SHD according to the intervention group before (*n* = 32) and after (*n* = 25) the intervention. Swedish names are presented in italics

Before the intervention				After the intervention			
Rank	Intervention group^a^	M*	SD**	Rank	Intervention group	M	SD
1	*Isa 25*	5.17	1.0	1	*Isa 25*	4.25	1.0
2	*Björn 45*	5.00	0	2	Lynn 25	4.00	1.6
3	*Karin 70*	4.25	1.5	3	Manuela 45	3.67	2.1
4	Elliot 25	3.75	1.0	4	*Johanna 45*	3.67	1.2
5	Urghesa 45	3.44	1.1	5	*Alexander 25*	3.40	0.9
6	Manuela 45	3.33	1.6	6	*David 70*	3.40	1.0
7	Lynn 25	3.33	1.2	7	Li-Xing 70	3.20	0.8
8	*Johanna 45*	3.25	0.5	8	Elliot 25	3.00	1.4
9	*Alexander 25*	3.00	0	9	*Björn 45*	3.00	0.8
10	Li-Xing 70	2.56	0.9	10	*Karin 70*	3.00	0.8
11	Ahmed 70	2.25	1.0	11	Urghesa 45	2.67	1.2
12				12	Ahmed 70	2.00	0

^a^There were no assessments concerning David 70 in the measurement before the intervention

*Mean

**Standard deviation

**Table 9:** Likelihood of having called SHD according to the control group before (*n* = 32) and after (*n* = 25) the intervention. Swedish names are presented in italics

Before the intervention				After the intervention			
Rank	Control group	M*	SD**	Rank	Control group	M	SD
1	Elliot 25	4.40	0.7	1	*Johanna 45*	4.88	1.1
2	*Isa 25*	4.25	1.9	2	*Alexander 25*	4.75	0.9
3	Lynn 25	4.25	1.0	3	*Isa 25*	4.00	0.7
4	*Alexander 25*	4.20	0.4	4	*Karin 70*	4.00	0.6
5	Manuela	4.00	0.8	5	Manuela 45	3.83	0.8
6	*Johanna 45*	3.67	1.4	6	Elliot 25	3.75	0
7	Urghesa 45	3.56	1.3	7	Li-Xing 70	3.60	0.9
8	*Björn 45*	3.33	0.6	8	*David 70*	3.55	0.9
9	*Karin 70*	3.33	0.8	9	Lynn 25	3.38	0.5
10	*David 70*	3.00	0	10	Ahmed 70	3.00	0.8
11	Ahmed 70	2.89	0.8	11	*Björn 45*	3.00	0
12	Li-Xing 70	2.50	0.9	12	Urghesa 45	3.00	0

*Mean

**Standard deviation

Finally, the participants were asked how likely it was that the fictive persons had been recommended a doctor’s appointment when they called SHD and in Tables 10 and 11 the results are presented.

**Table 10:** Ranking of likelihood of having got a doctor’s appointment according to the intervention group before (*n* = 32), and after (*n* = 25) the intervention. Swedish names are presented in italics

Before the intervention				After the intervention			
Rank	Intervention group^a^	M*	SD**	Rank	Intervention group	M	SD
1	*Isa 25*	3.83	1.3	1	Urghesa 45	4.00	2.0
2	Manuela 45	3.83	1.0	2	*Karin 70*	4.00	1.4
3	Urghesa 45	3.67	1.1	3	Manuela 45	4.00	1.0
4	Elliot 25	3.63	0.9	4	*Johanna 45*	3.67	1.2
5	Ahmed	3.63	0.9	5	Li-Xing 70	3.60	1.1
6	Li-Xing 70	3.56	1.2	6	*Isa 25*	3.50	1.7
7	*Karin 70*	3.50	0.6	7	*David 70*	3.50	1.0
8	Lynn 25	3.00	1.1	8	Elliot 25	3.50	0.7
9	Alexander 25	3.00	0.0	9	Ahmed 70	3.50	0.7
10	*Björn 45*	3.00	0.0	10	Lynn 25	3.25	1.3
11	*Johanna 45*	3.00	0.0	11	*Björn 45*	3.00	0.7
12				12	*Alexander 25*	2.80	0.4

^a^There were no assessments concerning David 70 in the measurement before the intervention

*Mean

**Standard deviation

**Table 11:** Ranking of likelihood of having got a doctor’s appointment when calling according to the control group before (*n* = 41) and after (*n* = 35) the intervention. Swedish names are presented in italics

Before the intervention				After the intervention			
Rank	Control group	M*	SD**	Rank	Control group	M	SD
1	*Johanna 45*	3.50	0.5	1	*Björn 45*	5.00	0
2	Li-Xing 70	3.10	0.8	2	Urghesa 45	3.25	0.5
3	Manuela 45	3.10	0.6	3	*David 70*	3.00	1.2
4	Ahmed 70	3.10	0.6	4	Manuela 45	3.00	0
5	Elliot 25	3.00	1.2	5	Li-Xing 70	3.00	0
6	*Björn 45*	3.00	1.0	6	Alexander 25	2.87	0.8
7	Urghesa 45	3.00	0.9	7	*Karin 70*	2.83	1.2
8	*Karin 70*	3.00	0.6	8	Lynn 25	2.75	0.9
9	*Isa 25*	3.00	0.0	9	*Isa 25*	2.60	0.5
10	Lynn 25	2.86	0.7	10	Elliot 25	2.50	0.6
11	*Alexander 25*	2.80	0.4	11	*Johanna 45*	2.38	1.1
12	*David 70*	2.50	0.7	12	Ahmed 70	2.00	1.4

*Mean

**Standard deviation

### Effect of the intervention

Every participant assessed two fictive persons before the intervention and two after the intervention. The same pairs of fictive persons were assessed in the intervention and the control group. In Table 12 the number of assessments is presented. The same assessment in both measurements indicated that the respondents assessed that the two fictive persons had been treated equally. Different assessments in the two measurements indicated non equal treatment. The answers in the two groups were almost the same, but with a slight tendency to fewer equal assessments for the questions about calling SHD and probable experience of discrimination.

**Table 12:** Number of assessments. The columns giving the same assessment indicate that the two fictive persons were treated equally and the columns giving different assessments indicate non equal treatment

Question	Intervention group				Control group			
	Before (*n* = 32)		After (*n* = 25)		Before (*n* = 41)		After (*n* = 35)	
	Same	Different	Same	Different	Same	Different	Same	different
Called SHD	12	20	7	18	13	18	17	18
Doctor’s appointment	25	7	20	5	34	7	27	8
Quality of life	21	11	17	8	24	17	21	14
Power over own life	16	16	11	14	20	21	19	16
Discrimination	15	17	8	17	18	23	16	19

### Evaluation of the seminar

A separate evaluation form of the educational intervention was distributed directly after the seminar and was completed by all seminar participants (*n* = 19). It included questions on how the participants valued the literature, the lecture and the seminar. Overall, participants were content with the intervention. 18 out of 19 had read the suggested literature and rated it as “very good” (2), “good” (8) or “rather good” (7). None rated it as “less good”, but two participants did not answer the question. All but two participants had watched the lecture. It was rated as “very good” by three participants, as “good” by nine and as “rather good” by two participants. None rated it as “less good”, but five did not answer. The perceptions of the seminar were all “very good” (9) or “good” (10).

## Discussion

The results showed few significant differences before and after the intervention in the intervention group. Also in the control group few significant differences were found in the two measurements, although no intervention was performed in that group. The reason for this might be that the instrument used was not sensitive enough to pick up an expected raised awareness concerning equity in healthcare or that solely the act of filling out the questionnaire (as was done in the control group) can create a sort of intervention effect, in that it can inspire the participants to reflect on issues related to equity in healthcare.

However, participants of the intervention group expressed that they appreciated the educational intervention, not least the seminar, where they got the chance to discuss equity in healthcare with two researchers and with engaged colleagues. The separate evaluation form, distributed directly after the educational intervention, further showed a high appreciation of it, concerning both form and content, indicating that telephone nurses need opportunities to discuss these issues and find them interesting and thought provoking.

The results indicate that awareness concerning equity in healthcare is difficult but important to study. This has previously been reported by Andersson et al. [55] in a Swedish context and by Verdonk et al. [56] in a Dutch context.

In the analysis, a Good life-index was created, consisting of assessments of experienced quality of life, power over one’s own life and experience of discrimination. The results showed that the fictive persons born in Sweden were assessed to have a higher Good life-index in all four assessment situations. This is in line with the societal context were the intervention was made, as previous studies and statistics have shown that people with foreign background are to a higher extent discriminated against than people born in Sweden by Swedish parents [54].

This was primarily obvious in the assessments of Alexander, who was described as a young man born in Sweden, and Li-Xing, who was described as an older woman born outside Europe. Before the intervention, no significant difference in the assessment of these two fictive persons was found, but after the intervention small differences were found, both in the intervention group and in the control group. The difference in the intervention group could be caused by the intervention. The same pattern in the control group could either be a coincident or an effect of the first questionnaire.

According to the theoretical frame of the study, ethnicity intersect with other factors, such as socio-economy, education, gender and age [27, 38, 42]. Concerning gender, the results showed no significant differences in the assessments of good life in the intervention group after the intervention. In the control group there was a significant difference in the evaluation made after the intervention. This could be interpreted as a higher awareness in the intervention group of the intersecting of different categories, making them distance themselves from easy connections between gender and inequality, and this might be a result of the educational intervention which covered aspects of intersectionality. When age was considered, significant differences were found in both groups. In short, younger age was assessed as improving the possibility of experiencing a good life.

Also the likelihood of the fictive persons having called SHD was assessed. The fictive persons with Swedish names were assessed to have called to a higher extent than the fictive persons with non-Swedish names. This is in line with previous studies of SHD and similar services [5, 16, 21]. Concerning the likelihood of having received a recommendation or referral to a doctor’s appointment, no significant differences were found before or after the intervention in any of the groups.

In order to measure the effect of the intervention, we compared the answers of the same persons before and after the intervention. The answers were almost the same, but with a slight tendency to fewer equal assessments for the questions about calling SHD and probable experience of discrimination. This might be an effect of the intervention, as a high awareness of equity in healthcare can concern awareness of discrimination based on gender, age or ethnicity, as well as the intersection of these factors.

Our results have practical implications in that both the questionnaire and the education developed within the project can be used to improve awareness of equity in healthcare in different settings. They can inspire discussions and change behavior, which might have direct impact on the encounter between healthcare provider and patient or caller. An increased awareness of what the law and different guidelines require when it comes to equity in healthcare are also likely to have positive outcomes on patients and callers, which in the long run can improve public health.

## Strengths and limitations

To our knowledge, this is the first intervention study of this kind. The study was made in Sweden on a rather small sample. We do claim, however, that the results are transferrable to similar contexts, i.e., Western countries with similar services as SHD. The factors that were measured were gender, ethnicity and age, but in future studies other aspects could be included, such as heteronormativity and dis/ability. Gender and ethnicity were indicated through the fictive names and information of birth country, which might be considered a weakness, but is based on how telephone nurses encounter persons that use the service.

The mean age of the studied population can be regarded as quite high, as it was over 50 in all groups. However, the sample reflects the mean age of telephone nurses in Sweden, as experienced nurses are valued within this field. This has previously been pointed out by Kaminsky et al. [8].

The intervention had a solid theoretical base and the results were interpreted by a multi-disciplinary group of researchers (two RN:s, one ethicist and one psychologist). Further, principles of adult learning [49, 50] were considered when designing the educational intervention. However, the instrument used for evaluations before and after the intervention is still under development and solely captures self-reported results by the participants. In order to investigate long term impact and outcomes, new measurements are required, as well as investigation of outcome of calls, or analyses of authentic calls.

## Conclusion

The results showed that fictive persons born in Sweden were assessed to have a higher Good life-index than fictive persons born outside Europe in all four assessment situations. Also younger age was assessed as improving the likelihood of having a good life, irrespectively of the educational intervention. The results are an imperative that equity in healthcare still needs to be educated and discussed in different settings. The encounter between telephone nurse and caller can impact the following care, which make the awareness of equity in health among telephone nurses an urgent matter. The intervention and the questionnaire used in our study were designed to fit telephone nurses. They could, however, easily be adjusted to other professional groups, who to an equal extent need to increase their awareness of equity in healthcare.

## Abbreviations

CDA: Critical discourse analysis
NPM: New public management
SHD: Swedish healthcare direct
